# Predominant role of Tax sumoylation in Tax-induced NF-kB activation in T cells

**DOI:** 10.1186/1742-4690-8-S1-A132

**Published:** 2011-06-06

**Authors:** Amandine Bonnet, Arnaud Favre-Bonvin, Patrycja Nzounza, Martine Nedelec, Maxime Chazal, Laetitia Waast, Voahangy Randrianarison, Ali Bazarbachi, Renaud Mahieux, Laurence Benit, Claudine Pique

**Affiliations:** 1 INSERM, U1016, Institut Cochin, Paris, 75014, France; 2 CNRS UMR 8104, Institut Cochin, Paris, 75014, France; 3 Univ Paris Descartes, Paris, France; 4 Department of Internal Medicine, American University of Beirut, Beirut, Lebanon; 5 INSERM, U758, Ecole Normale Supérieure de Lyon, Lyon, France

## 

Tax is a powerful activator of the NF-kB pathway, a property that is required for HTLV-1-induced T cell immortalization. Tax activates the NF-kB pathway by acting both at cytoplasmic and nuclear levels. In the cytoplasm, Tax binds to and activates the IkB Kinase (IKK) complex while in the nucleus, Tax assembles transcriptional active nuclear bodies. Others and we have previously demonstrated that the cytoplasmic/nuclear partition and NF-kB activity of Tax critically depend on its post-translational modifications. NEMO binding and IKK activation in the cytoplasm depends on Tax ubiquitination while Tax SUMOylation facilitates Tax nuclear body formation. Based on these findings, the current view is thatTax ubiquitination and SUMOylation cooperate to ensure optimal NF-kB activation by successively regulating the cytoplasmic and nuclear events. However, many questions remain regarding the individual properties of ubiquitinated or SUMOylated Tax and how the intracellular trafficking of Tax is coordinated to NF-kB activation. To explore these issues, we took advantage of the isolation of new Tax mutants that are ubiquitinated but poorly SUMOylated. We found that lack of SUMOylation modifies neither Tax stability nor Tax ubiquitination. In addition, while absence of SUMOylation prevents Tax nuclear body formation, this does not preclude Tax import into the nucleus. Finally, absence of SUMOylation reduces the NF-kB activity of Tax by around 70% in T cells. We are currently investigating the effect of fusion to SUMO isoforms on the activity of the mutants. Based on these new findings, we will propose a refined model for Tax-induced NF-kB activation in T cells.

